# A real-time system for selectively sensing and pacing the His-bundle during sinus rhythm and ventricular fibrillation

**DOI:** 10.1186/s12938-020-00763-6

**Published:** 2020-04-10

**Authors:** Ankur R. Shah, Muhammad S. Khan, Annie M. Hirahara, Matthias Lange, Ravi Ranjan, Derek J. Dosdall

**Affiliations:** 1 Department of Biomedical Engineering, The University of Utah, Salt Lake City, UT 84112 USA; 2 Nora Eccles Harrison Cardiovascular Research and Training Institute, The University of Utah, Salt Lake City, UT 84112 USA; 3 Division of Cardiovascular Medicine, Department of Internal Medicine, The University of Utah, Salt Lake City, UT 84112 USA; 4 Division of Cardiothoracic Surgery, School of Medicine, Department of Surgery, The University of Utah, Salt Lake City, UT 84112 USA

**Keywords:** His-bundle, Sensing, Pacing, Multi-electrode array, Cardiac electrophysiology, Ventricular fibrillation

## Abstract

**Background:**

The His–Purkinje (HP) system provides a pathway for the time-synchronous contraction of the heart. His bundle (HB) of the HP system is gaining relevance as a pacing site for treating non-reversible bradyarrhythmia despite limited availability of tools to identify the HB. In this paper, we describe a real-time stimulation and recording system (rt-SRS) to investigate using multi-electrode techniques to identify and selectively pace the HB. The rt-SRS can not only be used in sinus rhythm, but also during ventricular fibrillation (VF). The rt-SRS will also help investigate the so far unknown causal effects of selectively pacing the HB during VF.

**Methods:**

The rt-SRS consists of preamplifiers, data acquisition cards interfaced with a real-time controller, a current source, and current routing switches on a remote computer, which may be interrupted to stimulate using a host machine. The remote computer hosts a series of algorithms designed to aid in identifying electrodes directly over the HB, to accurately detect activation rates without over-picking, and to deliver stimulation pulses. The performance of the rt-SRS was demonstrated in seven isolated, perfused rabbit hearts.

**Results:**

The rt-SRS can visualize up to 96 channels of raw data, and spatial derivative data at 6.25-kHz sampling rate with an input-referred noise of 100 µV. The rt-SRS can send up to ± 150 V of stimuli pulses to any of the 96 channels. In the rabbit experiments, HB activations were detected in 18 ± 6.8% of the 64 electrodes used during VF.

**Conclusions:**

The rt-SRS is capable of measuring and responding to cardiac electrophysiological phenomena in real-time with precisely timed and placed electrical stimuli. This rt-SRS was shown to be an effective research tool by successfully detecting and quantifying HB activations and delivering stimulation pulses to selected electrodes in real-time.

## Background

The His–Purkinje system provides a pathway for the time-synchronous contraction of the heart [1]. The electrical impulses originate from the sino-atrial node of the heart, spread through the atria, progress through the atrioventricular node, the His-bundle (HB) to the left and right bundle branch, and activate the ventricles through the Purkinje fibers [1]. In recent years, HB-pacing is being considered as the most physiological site to pace and treat certain irreversible bradyarrhythmia [2, 3]. However, there are very limited tools to assist in identifying the location of HB [3].

The penetrating HB is located inferiorly and leftwards from the AV node and is usually accessed from the right atrial septum above the tricuspid valve [1, 4, 5]. In rabbit hearts, the penetrating HB is accessible on the surface of the right atrial septal wall [5]. However, in human and similar sized hearts, the HB is located 1-2 mm away from the surface [1]. In the clinic, the penetrating HB is accessed using a single helical-shaped electrode in a catheter through venous access [4]. The helically shaped screw electrode fixates in the atrial septal wall like a screw [6]. Identifying the location of HB is challenging because the HB is situated inconsistently in normal hearts and even more inconsistently in diseased hearts [7]. Therefore, better mapping tools are needed to identify and pace the HB selectively.

Another potential application for selective HB-pacing could be during ventricular fibrillation (VF). VF is the most frequent initial rhythm witnessed in cases of sudden cardiac arrest (SCA) [8]. SCA leads to a reduction or stagnation of cardiac flow, and thus under-perfuses and irreversibly damages vital organs of the body [8]. The likelihood of surviving SCA decreases by 10% every minute [8, 9]. In cases of SCA due to VF, the heart needs to be immediately defibrillated to bring it back to normal sinus rhythm [8]. Patients at risk of cardiac arrest are likely to receive an implantable cardioverter–defibrillator (ICD), which can automatically detect arrhythmias and respond to them with life-saving defibrillation shocks. Approximately 150,000 patients receive ICD implantations each year in the USA alone [10].

Patients experiencing an ICD discharge of 0.4 J of energy report the shocks to be painful [11]. When ICDs detect VF, they commonly use 25–35 J of energy to shock the heart and is very painful for the patient [11]. Such high-energy shocks also cause electroporation in the heart tissue around the location of the electrode, which can lead to disturbance in the conduction and function of the heart [12]. Even inappropriate shocks, or shocks not delivered due to a life-threatening arrhythmia, lead to increased mortality in patients with ICDs [13].

The basic principle of defibrillation in response to VF is to shock and simultaneously depolarize at least 75% of the heart to render the cardiac tissue unexcitable [14]. Currently, ICD leads are deployed at the right ventricular apex and have a large defibrillation threshold. Even though the right ventricular apex lead location is not the most efficient for defibrillation, it is the most convenient. One of the earliest and perhaps the only improvement clinically adopted relates to the shape of the defibrillation waveform. Biphasic waveshapes have significantly lower defibrillation thresholds as compared to monophasic waveforms [15].

Alternate strategies for lowering defibrillation thresholds have been investigated extensively. One class of alternative lower energy defibrillation strategy uses multiple electrodes placed across the heart, and each electrode is delivered with lower energy shocks to shock and depolarize 75% of the heart mass simultaneously [16]. However, implanting multiple electrodes across the heart is clinically problematic [17].

Another class of low-energy defibrillation strategy relies on a series of pulses instead of shocks for defibrillation. These approaches fall under two categories: [1] series of low-energy pulses delivered at or below the intrinsic VF cycle length [18–20]. A series of low-energy pulses in the form electric field gradient has shown to be effective in smaller animal heart simulation and experimental studies [19, 21, 22]. In practice, the electric field requirement at the location of the electrode remains high [19]. (2) High-frequency stimulation pulse, which can disrupt and halt the VF wave-fronts [23, 24]. High-frequency stimulation is recommended at strategic locations to halt VF wave-fronts [23]. However, there is no consensus about strategic locations [23].

In recent years, researchers have studied the role of the conduction system during VF. [25–27]. The HB is thought to be electrically linked to the Purkinje fibers through the conduction system during VF [25]. Purkinje fibers are thought to help maintain VF [26, 27]. Since the Purkinje fibers reach a large portion of ventricular tissue [1], and the HB is thought to be connected to Purkinje fibers during VF, pacing the HB could modulate or even terminate VF. However, to study such causal effects of HB-pacing during VF, we need a reliable way to sense HB activations rates during VF and respond to them time-precisely.

Our lab has shown that the HB can be detected with very high selectivity using an array of electrodes instead of a single electrode [25]. The goal of this study was to design, realize, and validate in explanted rabbit hearts a real-time stimulation and recording system (rt-SRS) with specifications meeting the requirements for [1] using multi-electrode arrays for accessing the HB and [2] identifying the HB activation rates even during VF and responding to them in real-time. This paper describes the real-time stimulation and recording system (rt-SRS) and all the algorithms developed to selectively identify electrodes directly on HB, sense its activation rate and pace the HB during sinus rhythm as well as during VF. Later, the rt-SRS performance was tested in explanted rabbit hearts.

## Results

**Fig. 1:**
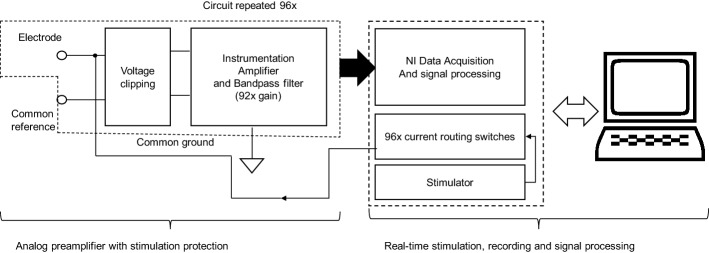
Block diagram of the complete 96-channel real-time HB sensing, recording, and stimulation system

The design of rt-SRS is sufficiently flexible to investigate multiple HB-pacing techniques. The rt-SRS consists of 4 main parts: (1) electrodes for stimulation/recording; (2) preamplifiers with voltage protection circuitry; (3) remote controller for time-precise stimulation/recording; (4) host machine for real-time visualization of raw electrograms and their spatial derivates, and to control the certain stimulation parameters on the remote machine (Fig. 1). Table 1 details all the specifications of rt-SRS hardware.

**Table 1:** System hardware specifications

96-channel preamplifier—(built inhouse)	
Preamplifier gain	162x
Input-referred noise	100 µV
Input impedance-between reference and each electrode	10^10^Ω
Frequency bandwidth	2.14 kHz
Bandpass center frequency, Q-factor	28.88 Hz, 0.013
SNR	69
Lower cut-off frequency, Q-factor	0.39 Hz, 1
Upper cut-off frequency, Q-factor	2.14 kHz, 0.5
96-channel real-time data-acquisition, recording and current routing hardware—(National Instruments, TX hardware modules assembled inhouse)	
Max sampling rate for each of 96 channels	6.25 kHz
Analog output channel configured for voltage to current converter	1 channel
Time to configure current source to a chosen channel	1.89 s
Time to trigger the switch after current channel configured	20 ms
Stimulator—(custom built by rdmApps, CO, USA)	
Stimulator	Unipolar, referenced to ground
Voltage to current conversion	±10 V to ± 10 mA
Output current range	±10 mA
Output frequency	0–10 kHz
Max impedance	10 kΩ

The pre-amplifier of the rt-SRS system amplifies the input signal by 162×, has a high-pass filter cutoff at 0.39 Hz, and a low-pass filter cutoff at 2.14 kHz. rt-STS can acquire and display the raw signal or spatial derivative (described later) of the raw signal in real-time at up to 6.25 kHz. The input impedance between each electrode and the reference was 10GOhm. The rt-SRS was tested in seven explanted Langendorff-perfused rabbit hearts. The HB signal could be recorded using the system with an average signal-to-noise ratio (SNR) of 69.

**Fig. 2:**
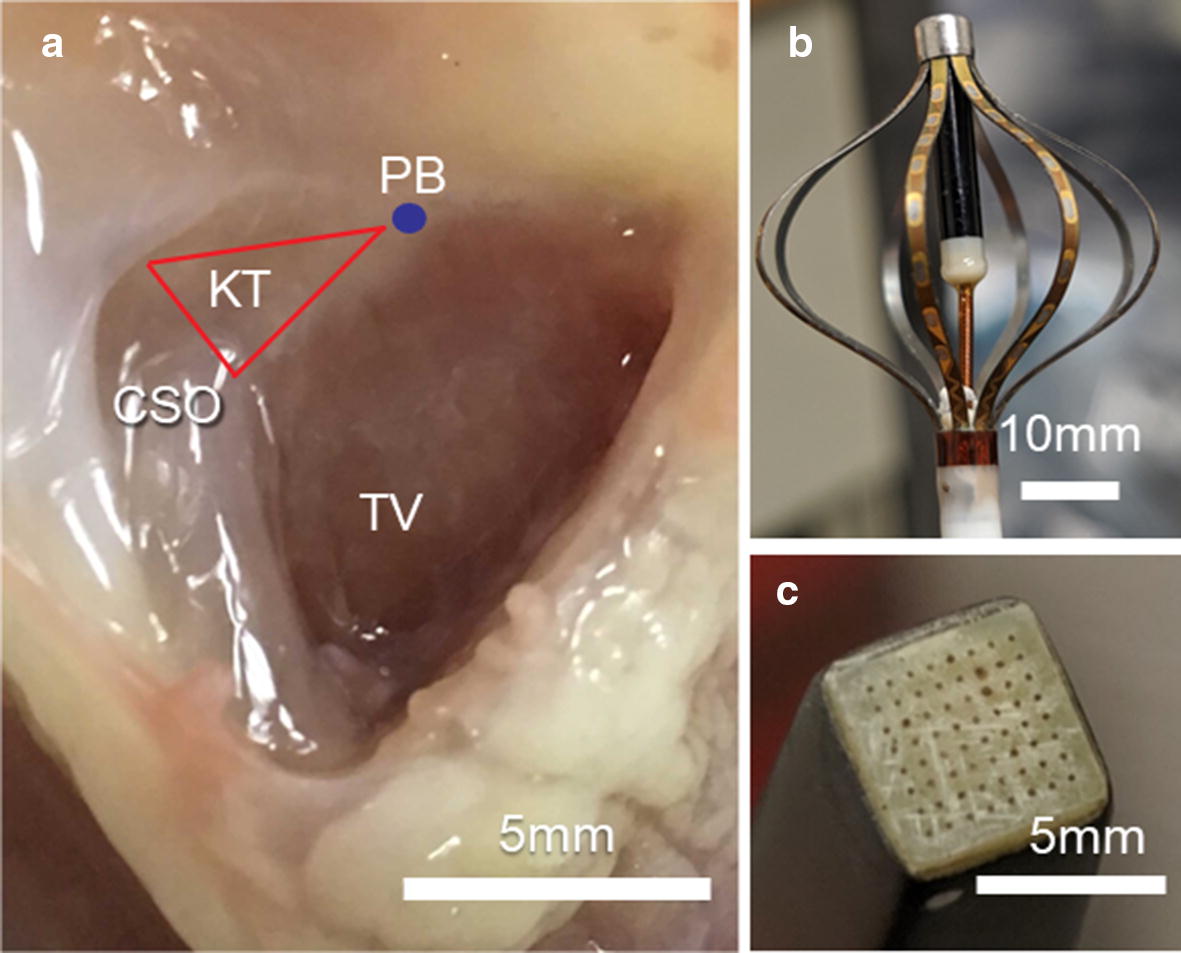
**a** Right atrial and upper ventricular septum of a perfused rabbit heart. *CSO* coronary sinus ostium, *KT* Koch’s triangle, *PB* penetrating bundle, *TV* tricuspid valve. **b** 64-channel Intellamap Orion basket catheter (Boston Scientific, Marlborough, MA), which was placed in the LV endocardium. **c** 8 × 8 MappingLab electrode array placed over the region of HB

The right atrium was incised from the opening of the inferior vena cava towards the right atrial appendage to expose the basal right ventricular septum, and the region containing the HB (blue dot, Fig. 2a) [28]. A flat 64-electrode array arranged in an 8 × 8 matrix (MappingLab Limited, Oxford, UK) (Fig. 2c for photo) interfaced with the preamplifier of the rt-SRS was placed over the region containing the HB. The number of electrodes on the array which have an HB signal is dependent on how the array is positioned at the target location. The atrial or ventricular signals were confirmed by time-correlating the atrial or ventricular signal on the pseudo-ECG (see “Methods”) with the atrial or ventricular signal deflections on the individual electrode’s raw or spatial derivative signal. If the observed deflection was in between atrial or ventricular deflection, it was determined to be HB because of the location where the electrode array was placed [28]. 50 ± 13.1% of the MappingLab array electrodes placed over the HB region (in n = 7 hearts) had a visible HB signal in the raw electrogram during sinus rhythm.

Spatial derivatives highlight the electrical activity underneath the central electrode by subtracting the common electrical activity between the surrounding electrodes [25, 29]. 27.2 ± 11.6% electrodes had HB signal in their spatial derivative, and 20 ± 11.6% electrodes had HB signal in the time derivative of the spatial derivative.

Later, the spatial derivates of the electrode array channels with the strongest HB signal were observed. Two channels with the strongest selective HB in the spatial derivative signal during sinus rhythm were chosen as candidates for selective HB sensing and pacing, respectively.

**Fig. 3:**
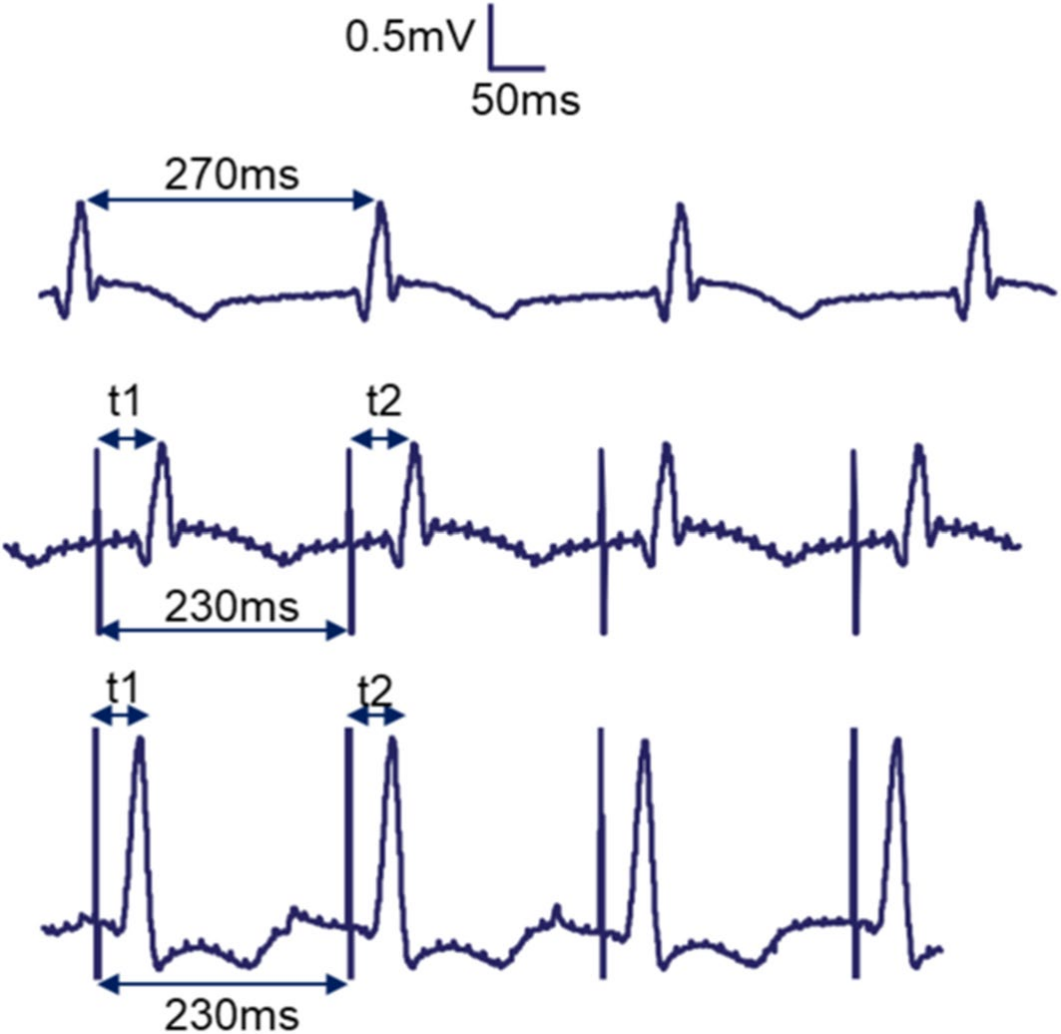
Pseudo-ECG traces during normal sinus rhythm (top), during HB-pacing with capture (middle), during VM-pacing and captured (bottom). In the top trace, the time interval between two QRS peaks (R–R interval) is 270 ms. When the HB was paced slightly faster than the intrinsic R–R interval with a current amplitude above the threshold, the shape of QRS around the peaks in the middle trace remained very similar to the shape of QRS around the peaks in the top trace. However, the R–R interval in the middle trace now adjusted to the rate of pacing. The stimulation artifacts in the pseudo-ECG in the middle trace are separated by 230 ms (shown). The time lag between the QRS peak and the stimulation artifacts also becomes constant so that t1 = t2 (shown). Similarly, when the VM was paced and captured, t1 = t2. However, unlike the top two traces, the shape of QRS is longer and different

If HB was captured, the atrial and ventricular rates on atrial and ventricular monitoring electrodes (see “Methods”) adjusted to the pacing rates, respectively (Fig. 3b, c). The shape of the QRS complex observed on the pseudo-ECG during sinus rhythm and during pacing when HB was captured were very similar (Fig. 3a, b). However, during pacing when the ventricular myocardium (VM) was captured, the QRS complex on the pseudo-ECG was wider and had a different morphology (Fig. 9b, c).

**Fig. 4:**
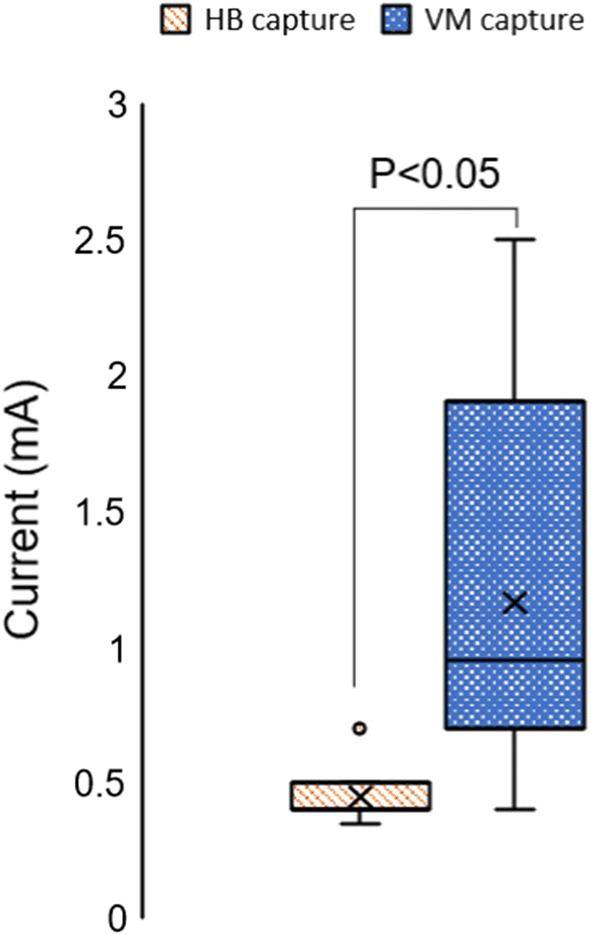
HB capture threshold vs VM capture threshold. The HB capture threshold was significantly smaller than the VM capture threshold (P = 0.033). Note that the standard deviation of the HB pace threshold was quite smaller than the VM pace threshold

During sinus rhythm, the current threshold for HB capture (0.46 mA ± 0.14 mA) was significantly lower than the threshold for VM capture (1.28 mA ± 0.71 mA) (Fig. 4). Note that the standard deviation for the HB capture threshold was also smaller than that of the VM capture threshold.

**Fig. 5:**
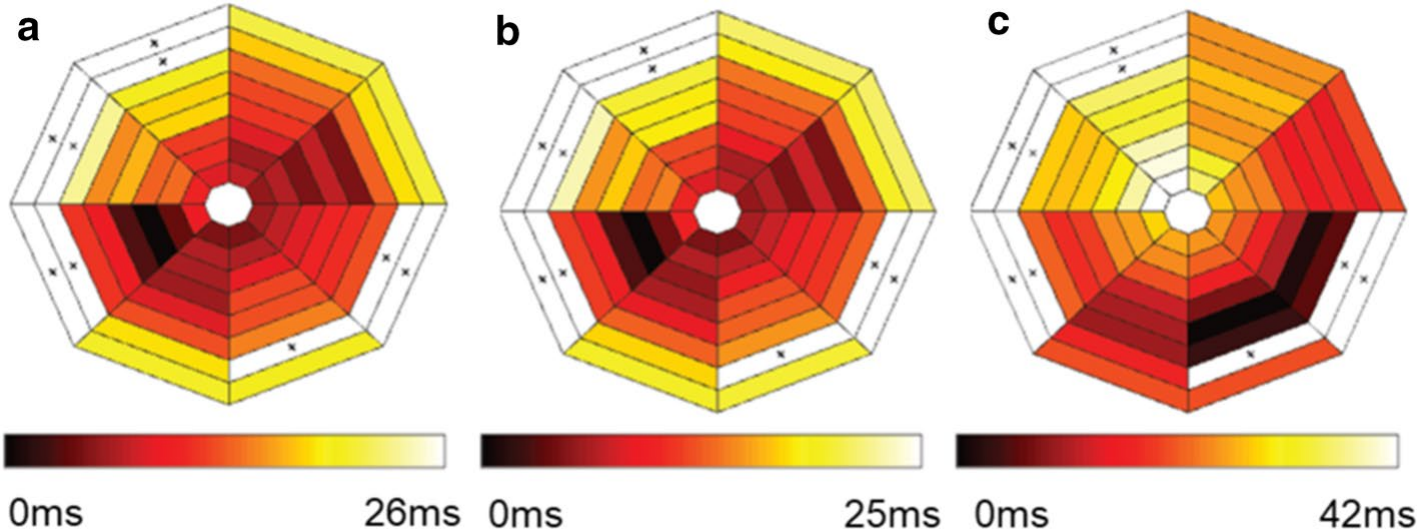
Activation maps from basket electrode array during normal sinus rhythm and pacing. Each plot has 8 splines with 8 boxes in each spline, and each box represents an electrode. If the electrode was broken or not in contact with the heart tissue, it was excluded from analysis and the respective electrode box in the above figure is identified by ‘x’. Darker color box indicates earlier activating electrode within a beat. Lighter colors represent later activating electrodes within the same beat. Boxes in the center are towards the apex, and the ones towards outside towards the base. **a** During sinus, **b** during sinus rhythm HB pace beat, **c** during sinus rhythm VM pace beat. Notice that A and B are similar and different from C

While HB or VM was paced, left ventricular (LV) endocardial electrical activity was recorded using a 64-electrode Orion mini-basket (Boston Scientific, Marlborough, MA) (Fig. 2b). The basket array was inserted through an incision made in the left atrium. During normal sinus rhythm, inside the LV of the heart, some regions activate earlier than other regions. Activations maps were created from basket electrode data to visualize which regions activated earlier rather than later. The ventricular endocardial pattern of activation recorded by the basket electrode array during sinus rhythm and when HB was captured looked very similar, and had a very similar time scale (~ 25 ms) (Fig. 5a, b, respectively). However when VM was captured the endocardial pattern looked very different and was longer (~ 25 ms in Fig. 5a, b vs. 42 ms in Fig. 5c).

After pacing the HB and VM during sinus rhythm, VF was induced in the rabbit hearts by pacing the LV free wall at 50 Hz for 30-s intervals until VF sustained. 18 ± 6.8% of electrodes had an HB signal during VF. All of the electrodes, which had the strongest HB signal in their raw signal as well as their spatial derivative signal, also had a distinct HB signal during VF.

In all the animals, sharp atrial signals were observed on a host of electrodes’ raw electrograms, and the atrial signal stood out on the corresponding electrodes’ spatial derivatives even during VF. Even though all electrodes had a ventricular signal in their raw electrograms during sinus rhythm, only three of the seven experiments had a ventricular signal in the time derivative of the spatial derivative of the electrodes. Of the electrodes which had ventricular signal in the time derivative of spatial derivative during sinus rhythm, only 15 ± 10.8% electrodes had a ventricular signal in the time derivative of spatial derivative during VF.

**Fig. 6:**
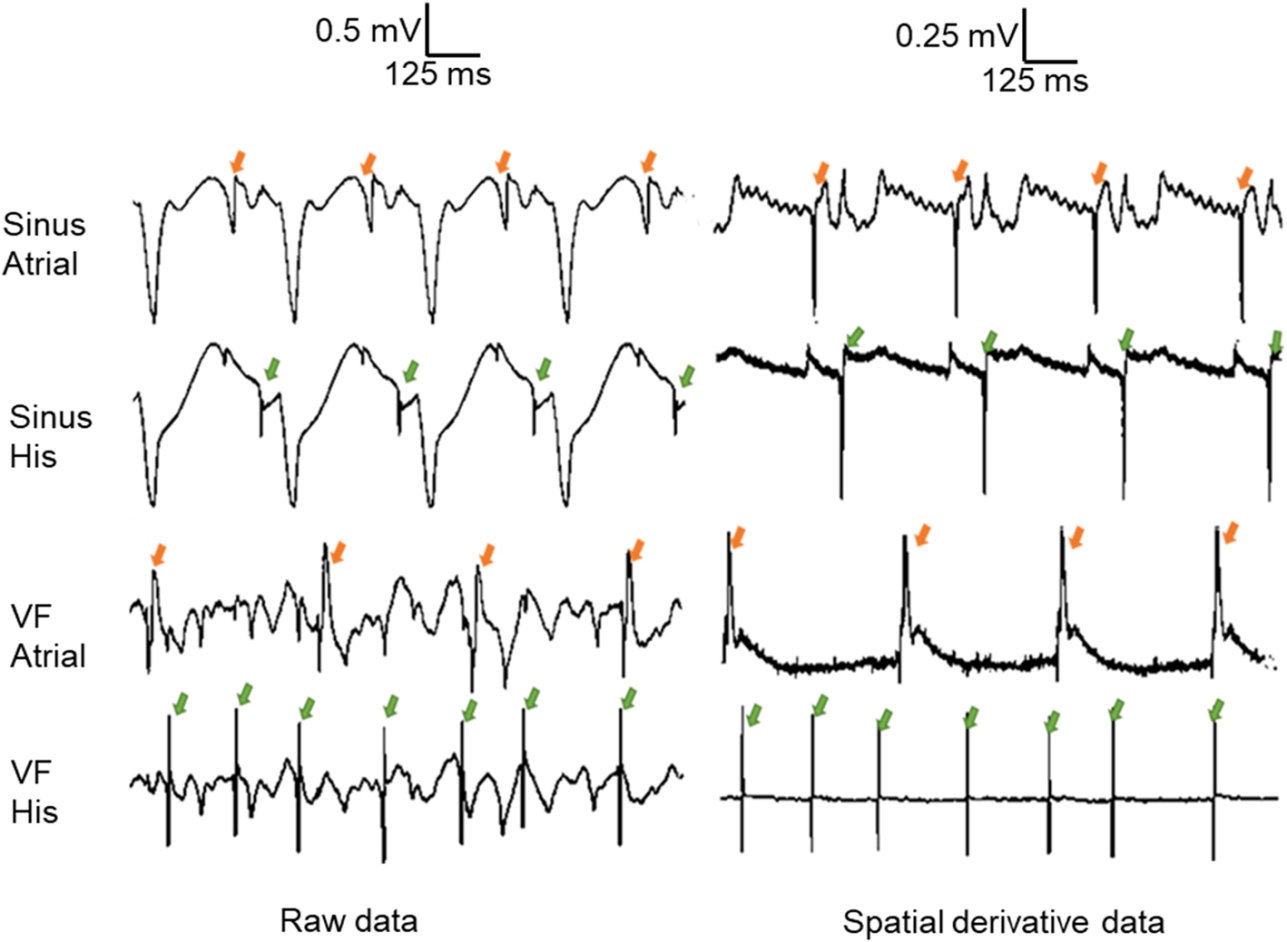
Selected traces with prominent atrial signal and prominent HB signal recorded using the real-time stimulation recording system. Arrows in orange point to atrial activations. Arrows in green point to HB activations. Left column: raw traces during normal sinus rhythm (top two) and VF (bottom two). Right column: spatial derivative traces during normal sinus rhythm (top two) and VF (bottom two). All atrial and HB traces are from the same respective electrodes

Figure 6 shows sample traces from one electrode with a strong atrial signal and another electrode with a strong HB signal during sinus rhythm as well as during VF. Traces on the left in Fig. 6 show raw electrograms, while the traces on the right in Fig. 6 show the spatial derivatives of the corresponding electrodes on the left over the same period. The top two traces have sinus rhythm data, and the bottom two traces have VF data. When the atrial signal had a sharp deflection during sinus rhythm in the raw electrogram for an electrode, the atrial signal gets prominently highlighted in its spatial derivative (see orange arrows in left vs. right top traces in Fig. 6). When the HB signal had a sharp deflection during sinus rhythm in the raw electrogram for an electrode, the HB signal gets prominently highlighted in its spatial derivative (see green arrows in left vs. right second to top traces in Fig. 6). The electrode, which prominently showed atrial signal in sinus rhythm spatial derivative data, also shows sharp deflections during VF in the raw electrograms. These atrial signals get prominent in the electrode’s spatial derivative signal (see orange arrows in left vs. right second from bottom traces in Fig. 6). Likewise, the electrode with a prominent HB signal during sinus rhythm shows deflections for HB signal even during VF. The HB signal became very prominent when the same electrode’s spatial derivative was observed (see green arrows in left vs. right in bottom traces of Fig. 6). The HB signal was so prominent that the HB activation rate could be easily detected using a simple thresholding algorithm (details provided in the section on software for rt-SRS).

## Discussion

HB can be paced with the least amount of current if the electrode is closest or ideally directly interfaced with the HB. Pacing tissue during ventricular fibrillation requires 5X–10X the current threshold required during normal sinus rhythm [30]. Since the stimulator power is limited, the lower pacing current threshold is useful. The rt-SRS can pace up to 10 mA for an electrode with impedance up to 10 kΩ across frequency spectra. Each channel’s amplifier is also protected for input voltages up to ± 150 V for any voltage surges, such as if the heart were to be defibrillated.

The rt-SRS described in this paper offers the following key advantages: (1) a convenient semi-automatic tool to detect electrodes directly over the HB; (2) real-time visualization of the raw electrograms and their spatial derivates from all the electrodes; (3) a real-time way to detect atrial, ventricular, and importantly HB activation rate during sinus rhythm as well as during VF; (4) real-time stimulation through an electrode of interest with hardware limiting time delay of 1.89 s for current route configuration and an additional switching delay of 20 ms.

Histology and microelectrode recording studies conducted in rabbit hearts have shown that the penetrating HB is directly accessible on the surface of the high right atrial septum [5, 28]. However, the VM surrounding the penetrating HB is underneath layers of the central fibrous body (CFB) tissue [5]. Very few electrodes had ventricular activations in the time derivative of the spatial derivative signal likely because the VM tissue is underneath CFB. Such ventricular activations were even more difficult to detect during VF. One likely reason why very few electrodes could detect distinct activations during VF is that the amplitude of activations decreases significantly during VF [31]. We inserted a separate wire electrode to pace the VM or detect the VM activation rate. Even though care was taken to insert the wire electrode at the same location, the insertion depth may have varied. Depending on where the subendocardial electrode was inserted, it may have influenced the pacing thresholds. If the VM was being captured through CFB tissue, high current levels may be expected as compared to an electrode directly pacing the VM tissue. This may explain why we saw larger standard deviation in VM capture thresholds as compared to HB capture threshold. Since the HB is located on the surface the capture threshold for HB was also significantly lower than the capture threshold for VM (Fig.  4).

For validating that the HB was indeed paced during experimentation, pseudo-ECG was used during the experiment (like in Fig. 3) and the LV endocardial basket data (like in Fig. 6) later confirmed HB or VM-pacing in more detail after the experiment. In Fig. 3, the top (no pacing) and middle (HB-pacing) traces, the QRS complex looks strikingly similar and certainly different from the bottom trace (VM-pacing). During sinus rhythm without pacing, the heart contracts regularly because of the heart’s intrinsic pacemaker, and the activations spread across ventricles through the conduction system of which HB is a part. While pacing HB, the heart’s regular beats are taken over by the pacing instead of the pacemaker, and the conduction still goes through the conduction system. Since in either no-pacing or during HB-pacing the conduction system is the primary pathway, the QRS complex looked strikingly similar in the top two traces of Fig. 3. However, while pacing VM, the activation does not spread across ventricles through the conduction system but using the lesser efficient ventricular tissue. Therefore, the QRS complex in the bottom trace of Fig. 3 is broader and also looks strikingly different. A more detailed response to either location of pacing was sought from a 64-channel basket electrode inserted in the left ventricle. Figure 5a was extracted from a beat during normal sinus rhythm without pacing, while Fig. 5b was extracted from a beat during normal sinus rhythm while pacing the HB. Notice that Fig. 5a, b are strikingly similar in pattern as well as duration (~ 25 ms). However, in Fig. 5c, which was extracted from a sinus beat during VM-pacing, the pattern is visibly different and the duration is also longer (42 ms).

The features of the rt-SRS present the possibility of configuring it to study HB-pacing response in different ways. In one study, the rt-SRS was configured to study the effect of pacing location, pacing rate, and the interaction of pacing location and pacing rate in early VF while perfusing the heart normally. In another study, the rt-SRS was reconfigured to study the effect of pacing the HB at a rate proportional to the ventricular endocardial activation rate in un-perfused VF over a while. The rt-SRS can also be configured to record electrical activity from all electrodes except the one through which stimulation pulses are sent. The rt-SRS can also be configured to send stimuli at precise time and rate.

## Conclusions

We developed a flexible system that meets the requirements to: (1) investigate using multi-electrode techniques to identify and selectively pace the HB; (2) study the effect of HB-pacing during VF in explanted hearts. The system was successful in detecting, quantifying, and responding to HB activations in real-time.

## Methods

### Electrodes

The excitability of tissue is sensitive to the stimulus current amplitude and waveshape [32]. The lower the impedance of electrodes interfacing with the tissue, the smaller the power requirement will be for a similar current amplitude. Silver/silver chloride electrodes were interfaced with the rt-SRS preamplifier as they have relatively low impedance, are good transducers of biopotentials, and also relatively non-polarizable [33]. A MappingLab electrode array with 64 electrodes arranged in an 8 × 8 matrix was interfaced with the preamplifier of the rt-SRS. Each electrode on the MappingLab array is made of silver, has a diameter of 0.1 mm and 0.43 mm spacing between two adjacent electrodes (Fig. 2c for photo). Before every experiment, the electrodes were chloridized if required so that all the electrodes had impedance under 10 kΩ using previously established procedures at the institute [33].

### Analog preamplification and voltage protection circuitry

**Fig. 7:**
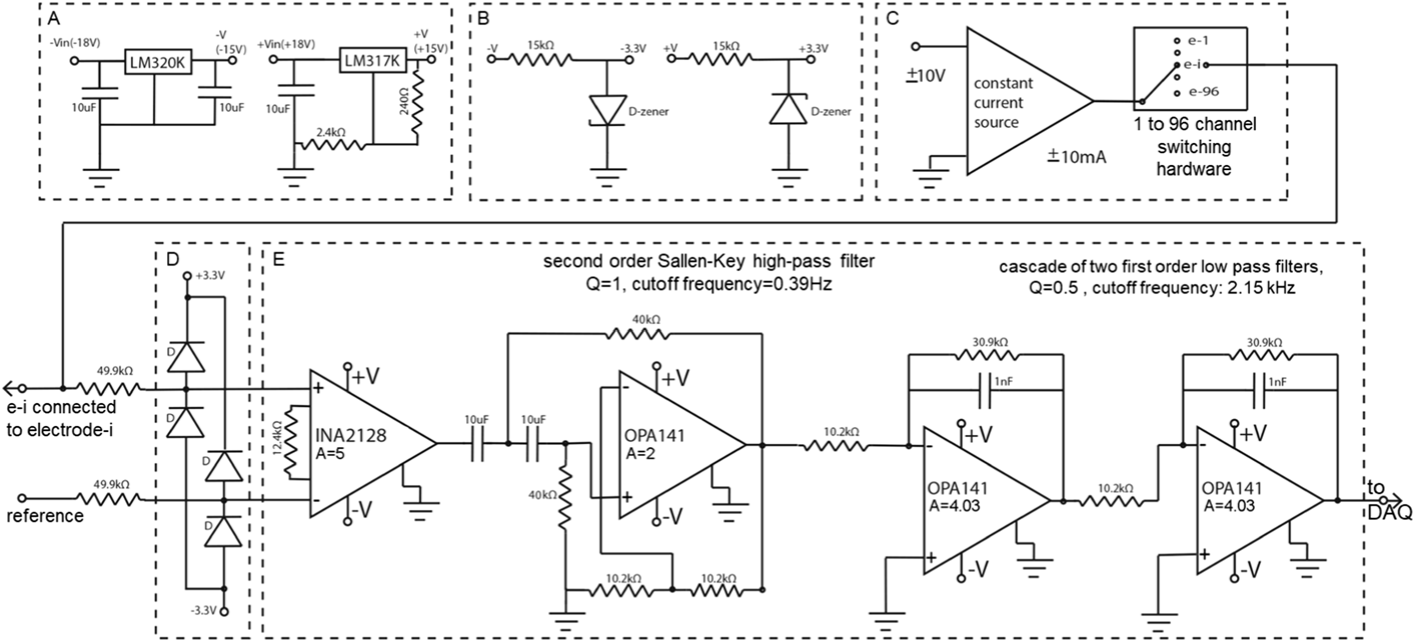
Preamplifier design **a** regulators to drop +18 V to 15 V. **b** Zener diodes used to drop 15 V to 3.3 V, which provides the reference voltage for voltage clipping circuit. **c** Voltage-to-current converter interfaced with a computer-controlled reed relay switching hardware. The current source signal could be routed to any of the up to 96 channels. **d** Voltage protection/clipping circuit. **e** Instrumentation amplifier, amplification, and filtration stages

The analog preamplifier clips any signal outside ± 3.3 V transduced by the electrodes, then amplifies and filters signals before digitizing them (Fig. 1). All the electronics were assembled on custom-built printed circuit boards (PCBs). Each PCB had preamplifiers for eight channels. A standard cage housed 12 PCBs with a custom backplane and battery rack to power the 96 preamplifier channels. Two 18-V 5.0-Ah rechargeable batteries (Makita BL1850B) were used to power the preamplifiers and provide ± 18 V. When the batteries powered all 96 channels of preamplifiers, they consumed 760 mA current, and the batteries lasted for about 6.5 h on one full charge. Two voltage regulators (LM317K and LM320N, Fig. 7a) were used to provide a constant ± 15 V power supply to the instrumentation amplifiers and the operational amplifiers. Two Zener diodes (MM3Z3V0ST1G) were used to drop ± 15 V to ± 3.3 V (Fig. 7b) and provided the reference voltage to the voltage clipping block.

The input of the preamplifier, in addition to interfacing with the electrode, interfaces with the current source through current routing instrumentation (see Fig. 7c). A constant current source converter converts any waveshape of ± 10 V (with frequency spectra 0-10 kHz) to ± 10 mA of the same waveshape. The current source provides up to ± 150 V. The ± 150 V limit implies that if the electrode impedance were 20 kΩ, the stimulator could source a maximum of 7.5 mA through that electrode. The stimulator was custom designed by RDM-Apps, CO, USA. A computer-controlled ‘reed relay switching matrix module’ is used to route the current signal to any of the 96 channels (see Fig. 7c).

The voltage at the electrode tips can rise to ± 150 V, especially while pacing. The preamplifier’s instrumentation amplifier (INA2128, Fig. 2d) may be damaged if exposed to voltages greater than ± 40 V. A voltage clipping circuit preceding the instrumentation amplifier clips any electrode signal bigger than ± 3.3 V. The voltage clipping circuit for each channel consists of 4 general-purpose diodes (CMDD3003TR, Fig. 7d). Each channel’s instrumentation amplifier interfaces with the electrode and the reference signal and provides a gain of 5× with high common-mode rejection to the input signal. The signal gets high-pass filtered through a 2nd-order Sallen Key filter with a cut-off frequency set at 0.39 Hz, and Q-factor of 1. The signal gets low-pass filtered through 2nd-order low-pass filter with a cut-off frequency set at 2.14 kHz, and Q-factor of 0.5 (Fig. 7e). The bandpass filter and the instrumentation amplifier provide an overall gain of 162×. The overall preamplifier bandwidth is 2.14 kHz, with a center frequency at 28.88 Hz, and a Q-factor of 0.013. The preamplifier’s input-referred noise is 100 µV.

### Data acquisition, processing, real-time visualization design

The output of each preamplifier channel connects to a channel of the data acquisition card. Two NI 6345 (National Instruments, Austin, TX) data acquisition cards are used to acquire up to 96 channels of data at 4096 Hz at 16-bit resolution. Of the 96 channels, one NI 6345 module converts 80 channels of preamplified analog data to digital data, and the second NI-6345 module converts the remaining 16 channels of analog data to digital signals. Each NI 6345 module can sample multichannel data at a maximum rate of 500k sample/second. Therefore, the rt-SRS can acquire data at up to 6.25 kHz per channel. The computer-controlled ‘reed relay switching matrix module’ uses three NI 2834A (National Instruments, Austin, TX) switches, which are stacked together to route constant current signals to any of the 96 channels (Fig. 7c). A NI-8840 (National Instruments, Austin, TX) remote controller establishes and handles communication with the data acquisition cards and current routing switches. A gigabit ethernet port on NI-8840 establishes the communication between the remote controller and a host computer. The host computer can interrupt the remote machine processes or provide a platform to visualize data the remote controller acquired in real-time.

### Software for rt-SRS

The data acquisition hardware and the current routing/switching hardware were organized using codes developed in LabView (National Instruments, Austin, TX). A host computer was connected to the remote controller through ethernet ports on both ends to deploy and monitor the software on the remote machine. LabVIEW programs are called virtual instruments (VIs) because the appearance and operation are similar to physical instruments like an oscilloscope or a multimeter. Every VI consists of a front panel and a block diagram. The front panel is the user interface of the VI and consists of controls and indicators. The controls are similar to the input to an instrument and feed into the block diagram of the VI. The indicators are similar to an instrument’s output device and display the data that the block diagrams acquire or generate. The block diagram consists of algorithms and functions implemented using a graphical representation. The block diagram acts upon the controls and displays the data generated or acquired on the indicators.

**Fig. 8:**
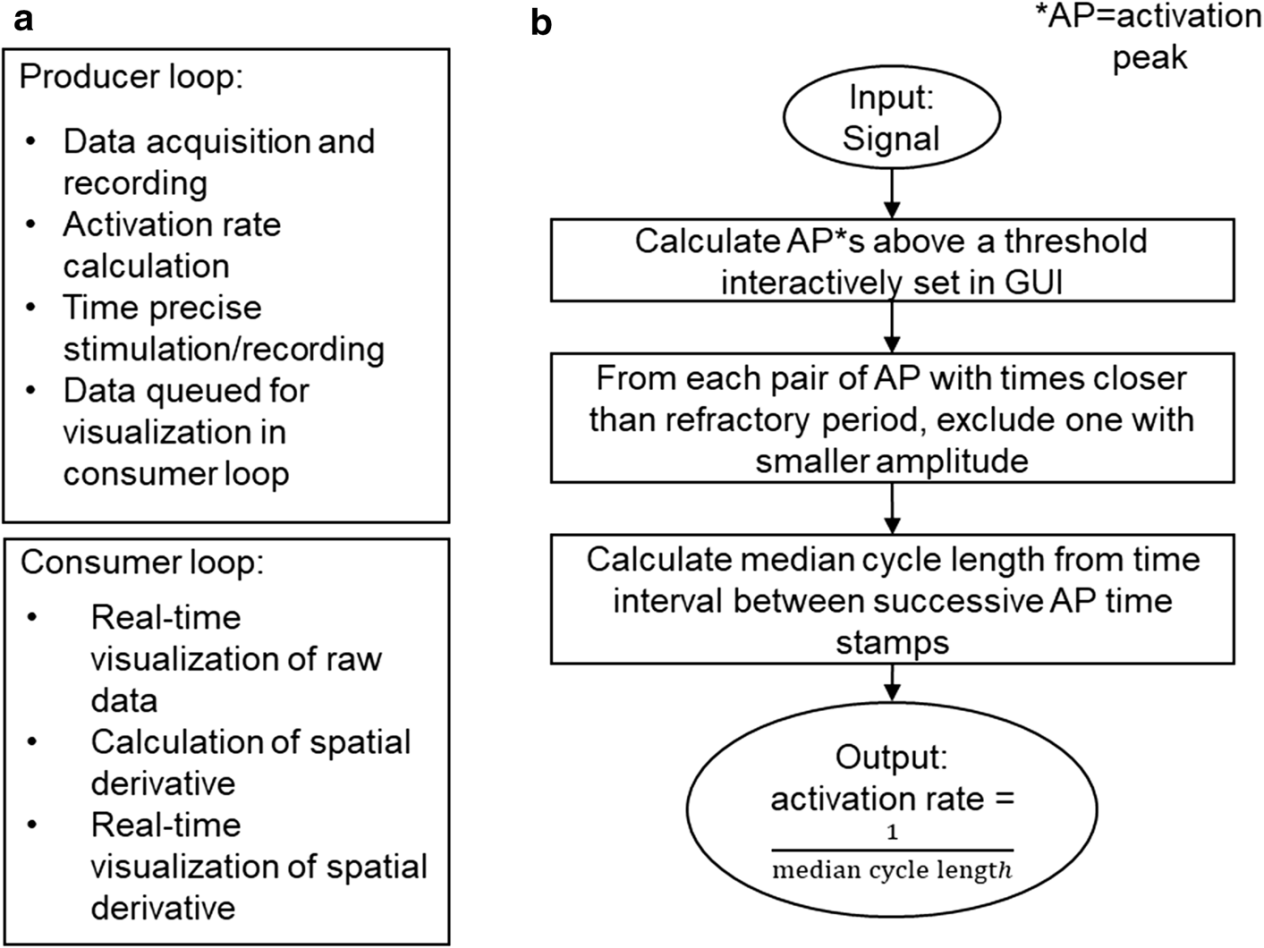
Description of software for rt-SRS. **a** The code for rt-SRS consists of two main loops. The producer loop handles codes for data acquisition, recording, activation rate detection, time-precise stimulation, and queues data for consumption in the consumer loop. The consumer loop handles codes for real-time display of raw data or calculation and real-time display of spatial derivative of the raw data. **b** Flow chart for choosing activations picked closer than a refractory period

The block diagram of our application has an event-structured producer/consumer design (see Fig. 8) [34]. The design consists of two main loops, namely the producer loop and the consumer loop. The producer loop of the block diagram has four ‘event’ states: initialize, data acquisition/recording, rate detection/stimulation, and stop. The consumer loop has two ‘event’ states: one to visualize the raw data in real-time and the other to calculate and visualize the spatial derivative of the raw data in real-time. Upon starting the VI, the producer loop starts in the initialize event state while one can initialize the controls for data acquisition sampling rate and data storage folder names. Other constants for data acquisition include voltage range, output voltage range for voltage-controlled constant current stimulator, data acquisition hardware, analog output hardware for voltage to current stimulator also get initialized while the producer loop is in the initialize event state. If any hardware configuration fails, the application will abort and show an error. After ascertaining the initialization controls, the data acquisition begins by clicking the start control button on the front panel. The producer loop is now in the data acquisition/recording state. The consumer loop is in the raw data state by default, but does not perform any function when the producer loop is not in the data acquisition/recording state. While the producer loop is in the data acquisition state, it acquires the raw data, records the data if the front panel record button is checked, and uses LabVIEW’s enqueue element feature to queue the data to a buffer for consumption in the consumer loop. Every time data gets queued in the producer loop, the consumer loop deques the enqueued data, unpacks each channel of electrical information, and displays it on a scrolling graph on the front panel. If the spatial derivative (described later) control on the front panel was selected, the consumer loop would be in the spatial derivative event state. Here, it not only unpacks each channel of electrical information, but also manipulates each channel’s electrical information using the other channel’s electrical information and displays this new information on a scrolling graph indicator on the front panel. The spatial derivative of electrical information is very useful in identifying electrodes directly on the HB.

Spatial derivatives highlight the electrical activity underneath the central electrode by subtracting the common electrical activity between the surrounding electrodes [25, 29]. The raw data or the spatial derivative data of up to 96 channels can be visualized in real-time on the front panel of the remote application on the host machine. When a matrix of the regularly spaced electrode array is used to measure electrical activity, the spatial derivate of an electrode is given by:

1 $$V{\text{c}} - \frac{{\left( {V{\text{w}} + V{\text{e}} + V{\text{n}} + V{\text{s}}} \right)}}{\text{count}},$$

where V is the voltage, subscript ‘c’ is for the central electrode, subscripts ‘w’, ‘e’, ‘n’, and ‘s’ are for electrodes west, east, north, and south of the central electrode. If the electrode has all the neighbors, then the count value is 4. However, edge electrodes have three neighbors, and corner electrodes have two neighbors. Therefore, the count value is 3 and 2, respectively, for the edge and the corner electrodes. Once the matrix of electrodes is placed over the region of HB, all the raw data channels are scanned for channels with the strongest HB signal. Later, the spatial derivates of the channels with the strongest HB signals are observed, and the channels with the strongest selective HB signal during sinus rhythm are chosen as candidates for selective HB stimulation. The spatial derivative of the electrode which has the strongest HB signal during sinus rhythm also has the strongest signal during ventricular fibrillation [25].

The producer loop of the block diagram enters the detection/stimulation event state upon selecting the sensing electrode, and the stimulation electrode controls on the front panel. The current routing hardware takes 1.89 s to configure the current route to the stimulation channel. Therefore, while in the detection/stimulation event state, the switching hardware gets configured while 3 s of data from the sensing electrode and its surrounding electrodes are acquired. An interactive graph on the front panel displays the spatial derivative of the sense electrode or the raw electrogram of the sense electrode. The graph has a horizontal line that can be dragged vertically to adjust the threshold to detect peaks. The peak detector function in LabVIEW was used to detect the intrinsic activation rate. Since the activation time coincides with a very sharp deflection, the peak width was set to 3 samples. During VF, the signal-to-noise ratio was lower for the LabVIEW’s peak detection function to accurately select the HB activations every time only using a threshold value and peak width value. Therefore, a custom function was built for cases when multiple peaks are detected, which are closer than a refractory period for cardiac tissue [35]. The refractory period is a duration within which another activation cannot occur. When the peak detector function detects multiple peaks, the custom function only keeps the biggest peak within the specified refractory period, and the algorithm excludes any remaining peaks. For experiments conducted in rabbit hearts, the refractory period was set to 30 ms [19]. If the threshold is set too close to zero, the peak detector function can choose too many peaks (> 50 picks per second), and the custom function will fail to remove the too many peaks within a reasonable time frame (< 2 s). Therefore, if the custom function fails to exclude over-picks within 2 s, the user can manually enter the activation rate or have a pre-assigned value to use as the pacing rate.

For a stimulation pulse to control cardiac tissue, it needs to pace at a rate faster than the intrinsic activation rate of the tissue [35]. Therefore, immediately after the HB activation rate was determined, current pulses were sent through the stimulation electrode at a rate of 5% faster than the intrinsic activation rate. The ability to pace immediately after detecting the HB activation rate is critical during VF as the activation rates vary a lot more during VF [25].

**Fig. 9:**
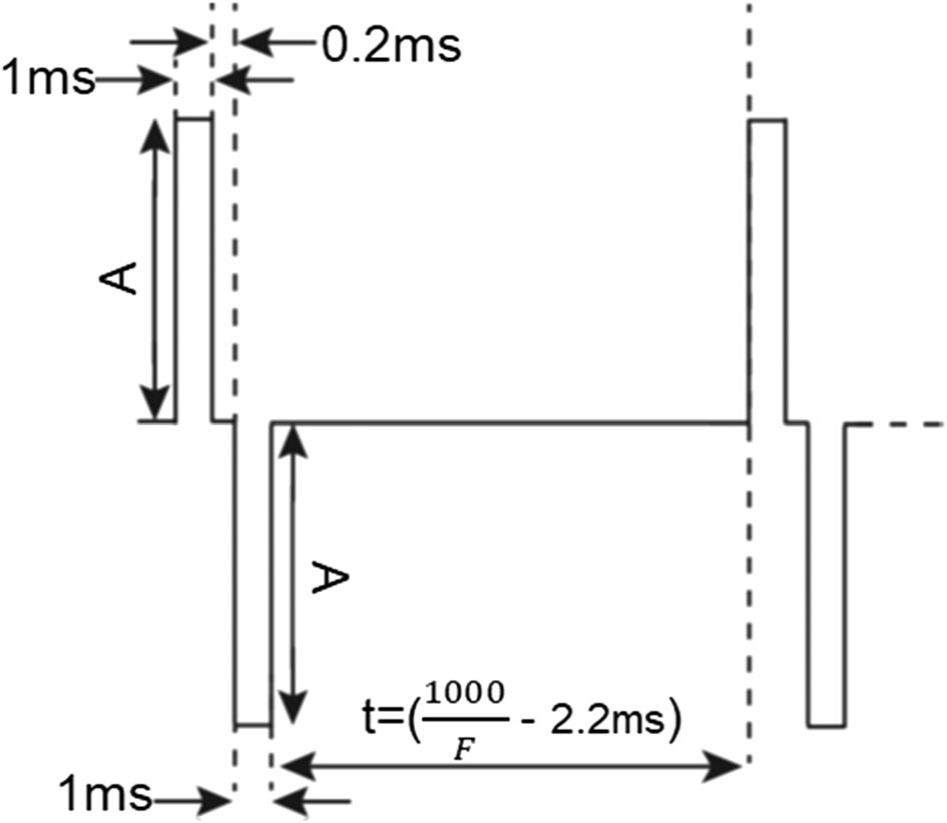
The basic waveshape used for pacing consisted of 1-ms positive phase, a 0.2-ms blanking period, and a 1-ms negative phase. The remaining blank period time (t) was determined by the frequency of pacing (F), which may be fixed or determined from the activation rate at an electrode. Amplitude (A) is the current amplitude and is adjustable

The stimulation waveform consisted of a 1-ms positive pulse, followed by a 0.2-ms blank period (or inter-pulse period), followed by a 1-ms negative pulse, followed by a blank period determined by the pace-rate (Fig. 9) [35]. If the pacing rate and the stimulation electrode were both set manually, and the rate detection algorithm were completely skipped, the rt-SRS takes 1.91 s (1.89 s to configure the current route and switching time was 20 ms) to deliver stimulation pulses after choosing the stimulation electrode.

## Experimental methods

### Heart preparation and Langendorff perfusion

The rt-SRS described was tested in seven explanted Langendorff-perfused rabbit hearts. All animals were handled in accordance with the Guide for the Care and Use of Laboratory Animals [36] and the protocol was approved by University of Utah’s Institutional Animal Care and Use Committee. Seven New Zealand rabbits were anesthetized with 40 mg/kg ketamine and 4 mg/kg xylazine. Half the drug mixture was delivered intramuscularly. The remaining half and 2000 IU of heparin were delivered intravenously. After the animal was in a deep surgical plane of anesthesia, a median sternotomy was performed, and the heart was excised and submerged rapidly in 4 °C Tyrode’s solution. Any blood or non-heart tissue along with the excised heart was removed in the cold Tyrode’s solution, and then the heart was Langendorff-perfused with 37 °C Tyrode’s solution. The Tyrode’s solution’s composition was as follows, in mmol/L: 4 KCl, 1.8 CaCl_2_, 130 NaCl, 1.2 NaH_2_PO_4_, 1 MgCl_2_, NaHCO_3_ 20.8, dextrose 11, and 0.04 g/l bovine albumin. The flow rate was adjusted to maintain pressure at 60-70 mmHg. The Tyrode’s solution’ was aerated with O_2_ and CO_2_ to maintain a pH of 7.4 ± 0.1. This preparation kept the explanted heart stable for at least 2 h.

### Integrated instrumentation for monitoring the heart’s electrical activity

The electrical stability of the heart was monitored using three electrical signals: (1) pseudo-ECG measured in the bath; (2) bipolar atrial signal, and (3) bipolar ventricular signal. The three signals were monitored in real-time using LabChart^®^ software through a Powerlab 16/30 system (AD Instruments, Colorado Springs, CO, USA). The pseudo-ECG was monitored using three electrodes placed around the heart in the bath. One of the three electrodes acted as the common ground for all the three electrical signals. Two wire electrodes were inserted posterior to the aorta in the left atrium to monitor the atrial activity. Two wire electrodes were also inserted in the RV posterior lateral free wall to monitor the ventricular activity.

### HB and VM-sensing electrodes setup

The right atrium was incised from the opening of the inferior vena cava towards the right atrial appendage to expose the basal right ventricular septum, and the region containing the HB (blue dot, Fig. 2a) [28]. The 8 × 8 flat MappingLab electrode array described earlier (Fig. 2c) was placed over the region containing the HB. Two electrodes with the most prominent HB signal were identified from the host machine using the real-time visualization of raw electrograms and their spatial derivatives. The HB activation rate was detected using one of the two electrodes, while the other electrode was used to pace the HB. Another silver wire was inserted in the high right ventricular septum to be able to pace the VM close to HB.

### Left ventricular endocardial recording setup

An Orion mini-basket electrode (Boston Scientific, Marlborough, MA) (Fig. 2b) was inserted in the left ventricle (LV) through an incision made in the left atrium. The Orion mini-basket electrode array has eight splines with eight small electrodes on each spline. The surface area of each electrode is 0.4 mm^2^, and the center-to-center inter-electrode distance is 2.5 mm. Figure 2b shows the Orion basket when expanded, and the diameter at the equator is 18 mm. When collapsed, the length of the basket from the catheter shaft (end of white in Fig. 2b) to the basket tip is 27 mm. The basket array recorded the left ventricular endocardial electrical activity while pacing the HB and VM. Based on sampling rate recommendation from a previous study, the basket array data were recorded at 4096 Hz with 24-bit resolution (Active Two System, Biosemi, Inc, Amsterdam, Netherlands) [37].

During normal sinus rhythm, inside the LV of the heart, some regions activate earlier than other regions. To visualize which regions, as mapped by the basket electrode array activated earlier rather than later, activation maps were created. Similar activation plots using basket data have been created in the past [6, 38]. The center region of the activation map has electrodes towards the apex, and the electrodes towards the base are on the outside (see Fig. 6).

#### Steps for identifying electrodes on HB using rt-SRS

The pseudo-ECG signal acquired to monitor heart stability was sourced and displayed in real-time along with the data acquired by rt-SRS. The data on rt-SRS was acquired at 4096 Hz for sampling rate compatibility with LV endocardial basket electrical data. During sinus rhythm, the atrial and ventricular signals on the pseudo-ECG were time-correlated with rt-SRS signals to distinguish between atrial, HB or ventricular signals on the raw or spatial derivative electrograms. The HB signal is time-aligned between the atrial and ventricular signals. A set of electrodes with the strongest HB signals was identified after scanning electrograms from all the electrodes. Later, the spatial derivative signal of the selected set of electrodes was observed. An electrode was selected for HB sensing or pacing if and only if the electrode’s spatial derivative only showed a signal time-aligned with HB activation. Pacing an electrode with a spatial derivative containing two signals (i.e., HB and atrial or HB and ventricular) will result in non-selective HB-pacing. Such electrodes are thought to be at the edge of HB. Achieving this level of selectivity is critical for the detection of HB activation rates during VF.

After pacing the HB and VM during sinus rhythm, VF was induced in the rabbit hearts by pacing the LV free wall at 50 Hz at 30-second intervals until VF sustained.

### Signal-to-noise ratio (SNR)

The SNR of rt-SRS for HB signal was taken as the average of HB SNR calculated from sinus rhythm HB recordings of n = 3 animals. The SNR was calculated as the ratio of the power of HB signal recorded during sinus rhythm on the sensing electrode, to the power of input-referred noise taken over an equal duration as the HB signal. The power of the signal was calculated as the sum of the squares of each sample divided by the number of samples in each signal.

### Statistical analysis

A Student’s T-test with equal variances was conducted in JMP Pro 14.0 for statistical testing. Results are written as mean ± SD and expressed as significant when P < 0.05.

## Data Availability

The datasets used and analyzed during the current study are available from the corresponding author on reasonable request.

## Abbreviations

VM: Ventricular myocardium
HB: His-bundle
VF: Ventricular fibrillation
CFB: Central fibrous body
ECG: Electrocardiogram
PCB: Printed circuit board
